# Satisfaction of doctors with their training: evidence from UK

**DOI:** 10.1186/s12913-017-2792-0

**Published:** 2017-12-29

**Authors:** Simon Gregory, Chiara Demartini

**Affiliations:** 1 Health Education England, London, England; 2 Homerton College, Hills Road, Cambridge, CB2, 8PH England; 3 Department of Economics and Management, University of Pavia, Via S. Felice, 5/7, 27100 Pavia, Italy

**Keywords:** Training satisfaction, Workload, Supervision, Feedback, Supportive environment, NHS

## Abstract

**Background:**

This study considers the primary training environment factors affecting the satisfaction of doctors in training with their training.

**Methods:**

An OLS multiple regression analysis was performed on responses given by doctors in training (trainees) to General Medical Council (UK) National Trainee Survey annually from 2012 to 2015. Two different research models investigate the determinant of trainee doctor satisfaction. The first model includes clinical supervision, feedback, workload, and gender as explanatory variables. The second model adds supportive environment to the first model.

**Results:**

The GMC survey response rate is 97%. Our analysis shows the key factors that determine trainee satisfaction are strong clinical supervision, frequent and useful feedback meetings, an adequate workload and a supportive environment.

**Conclusions:**

It is suggested focus on clinical supervision, feedback, workload and supportive environment would increase trainee satisfaction, improve the quality of training and morale, and hopefully, therefore, the quality of care patients receive.

**Electronic supplementary material:**

The online version of this article (10.1186/s12913-017-2792-0) contains supplementary material, which is available to authorized users.

## Key messages

- The GMC National trainee survey can be used as a tool to consider individual clinical units but also to take a global look at postgraduate medical training.
- To enhance trainee satisfaction and the quality of training focus should be on:
  - The balance of workload and provision of adequate and appropriate environment; and
  - Ensuring high quality accessible clinical supervision and high-quality feedback.

## Background

UK Junior Doctors are amongst the highest paid in Europe with well-developed training programmes that are not found in many other nations yet their morale is reported to be at its lowest since 2001 with a “state of unease within the medical profession across the UK as a result of increased pressures on health and social care services” [14]. There are reports that low morale of doctors currently training in England is leading to them applying for GMC certificates of good standing to apply for work overseas [31]. This suggests that a focus on ensuring the satisfaction of doctors with their training and employment is key.

The morale of doctors in training is currently a major issue in England, with recent industrial action in which doctors in training across England went on strike. Primarily this was a contractual but in negotiations it was made clear that it was not only that, much of it was general dissatisfaction with their working lives [16]. Doctors conditions were as bad, if not worse, in the 1980s and 1990s without strike action [20]. Therefore, we ask, whether junior doctors are satisfied with their training? Doctors in training in the United Kingdom are appointed through national standardised selection processes into programmes that rotate them through posts in a number of care environments (e.g. hospitals or general practices). These are often high-pressure environments with high workload and variable levels of support. To achieve trainee satisfaction, high quality of education and training and ultimately good patient care, trainees need to feel valued and supported across their working lives and in their learning [5, 29]. The General Medical Council (GMC), the UK regulator of doctors and medical education, and its predecessor for postgraduate medicine, the Postgraduate Medical Education and Training Board (PMETB) have surveyed all doctors in formal postgraduate training programmes in the UK annually for the past thirteen-years [13]. In 2015 53,000 doctors participated in this survey. Of these 53,000 33% rated their experience as excellent and 44% as good and only 4% rated it as poor or very poor which would suggest the recently reported low morale does not relate to their training [2, 6, 13, 30]. Given the reports of low morale and consequent discussions [16] the authors sought to further consider the responses of the 2012–2015 respondents against our research question on the key factors that support training and therefore satisfaction with this. Prior research reported the relevance of trainee satisfaction because it has been found to be linked to enhanced knowledge [4] and outcomes of care [38]. This study asks whether workload, clinical supervision and feedback affect trainee satisfaction?

Review of previous literature indeed suggests that trainee satisfaction is likely to be correlated with a triad of elements [8]; appropriate workload (sufficient to learn but not oppressive to educational opportunities and to well-being), good supervision of practice (clinical supervision) and the receipt of timely, good quality feedback. This is consistent with work from within and out with medicine. Results from studies on the implementation of the Working Time Directive showed that a reduction in hours has translated into increased evening and night shifts, with a decrease in day-time training [7]. This change in training has generated reduced trainee satisfaction in both the UK and the US [8]. Results from 58 psychiatric trainees showed that they are more satisfied when assigned to one supervisor, rather than two supervisors [23]. A Canadian study demonstrated that mentorship is the first determinant of trainee satisfaction [39]. Moreover, framing positive feedback has been found to positively impact trainees satisfaction [28]. General workplace studies have shown that satisfaction and performance are linked to a balance of workload and support [1, 12, 15, 18, 33, 36]. Other studies looking at trainee satisfaction have found correlation with age [19], operative experience and training programme allocation [26], and innovative training approaches [3], but such studies have been limited by sample size or restriction to single specialty/ branch of medicine (such as only surgical trainees e.g. [32]). This study considers trainees across all UK postgraduate medical specialties testing the GMC trainee survey as the largest annual survey of postgraduate medical trainees.

## Methods

Analysis of the main determinants of trainee satisfaction has been conducted on the responses given by UK trainees to the GMC National Trainee Survey between 2012 and 2015. This survey has been adapted from previously available trainee surveys (e.g. [9, 32, 37]). The unit of analysis of this study is the individual respondent (trainee). In order to secure confidentiality of individual respondents, all those groups of observations with a magnitude of three respondents or less have been withdrawn from the dataset by the GMC. After this withdrawal the number of valid observations included into the analysed dataset is 173,652. The GMC uses a total population sampling strategy, across specialties, over time. The response rate for this study is 97% (when the less-than-three-observation groups are retained). Thus, non-response bias can be assumed as negligible [24]. The total number of respondents in each of the study years varied from 43, 775 to 53, 077 however as anonymity is preserved the number of unique respondents across years cannot be determined.

### Variable measurement

In order to effectively measure research variables an exploratory factor analysis (EFA) on the whole dataset has been performed [11]. We carried out principal component analysis (PCA) with Varimax rotation, which identified six valid constructs. Only constructs showing eigenvalues higher than one were retained [21].

The current generic and demographic final questionnaire of the GMC National Trainee Survey can be found at http://www.gmc-uk.org/2017_BN3_Annex_A_pdf.pdf_71025099.pdf (Additional file 1).

Research variables included into this study are divided into dependent – trainee satisfaction – and independent variables – clinical supervision, feedback, workload and supportive environment – (Additional file 2) as follows.

*Trainee satisfaction* is conceived as the main dependent variable for this study and captures the individual general satisfaction of the trainee in the post with their clinical teaching programme. It is a multidimensional construct including variables from *overall satisfaction,* and *adequate experience* made up of six items, which achieve satisfactory levels of communality (0.5 or above), namely quality of teaching, quality of experience, quality of clinical supervision, recommendation of the post to a friend, usefulness of the post for future career, confidence in the acquisition of the competences needed at a particular stage of the training programme and the practical experience received in a post.

*Clinical supervision* is a multidimensional variable aimed at capturing the extent to which the trainee feels (s)he is clinically supported during their training activity. It is measured by the mean value of four items, accessibility of senior support when requested, competence of the supervisor as perceived by trainees, being required to cope with problems beyond their competence and taking of consent for procedures for which they did not feel competent to do so.

*Feedback* is a multidimensional variable measuring the frequency and quality of the feedback received by trainees from their supervisor. Its value is given by the average of three items, frequency of feedback, personal progress meeting and performance assessment.

*Workload* captures the intensity of trainees’ work as well as their perceived level of stress due to excess of work. It is made up of four items, the perceived intensity of day work, perceived intensity of night work, working beyond contracted hours and the perception of tiredness.

*Supportive environment* is a multidimensional construct, the mean value of five items, overall supportive environment, fair treatment of staff, respect, confidence building and superior-subordinate openness. The items retained in the supportive environment construct were introduced to the survey in 2015. Therefore, the number of observations for supportive environment is lower compared to the overall dataset.

In this study *Gender* (Male v Female) is used as control variable. Gender is coded as 0 if the respondent is male, and 1 if female.

#### Descriptive statistics

Descriptive statistics include minimum, maximum, mean values and standard deviation for all the research variables. In order to provide the distribution of responses according to the ratings, we computed the frequency of each rating for each question. Moreover, we performed a zero order correlation analysis to check for both correlation between research variables and multi-co-linearity.

#### Empirical models

Research models have been tested by performing Ordinary Least Squares (OLS) multiple regression analysis on the surveyed sample. The two models have been tested in order to apply to the main research question. Model 1 analyses the effect of clinical supervision, feedback, workload and gender on trainee satisfaction. Model 2 includes supportive environment as independent variable also. Since supportive environment has been introduced in the survey since 2015, Model 2 has been tested on the observations referring to the 2015 survey only. This two-stage approach aims at identifying the main contribution of supportive environment on the variation of the variable Trainee satisfaction.

## Results

Descriptive statistics for the surveyed sample are provided in Table 1. Feedback, workload and supportive environment range from 0 to 100, whereas clinical supervision ranges from 9 to 100 and trainee satisfaction has a 20 to 100 range. Overall the level of satisfaction is high and above the 80% level. However, the high standard deviation flags that there is quite a large difference in respondents’ satisfaction. This is also the case for the other research variables, with workload showing an even worse situation in terms of both mean value and in magnitude of the standard deviation. Nonetheless, the highest value of standard deviation is associated with feedback, where a further investigation on the reasons for such variation is strongly suggested. Table 2 reports frequencies for each item included into the research variables.

**Table 1:** Descriptive statistics of the research variables

*n* = 173,652	Mean	SD	Min	Max
Trainee satisfaction	80.929	14.477	20	100
Clinical supervision	90.015	11.672	6.25	100
Feedback	76.159	22.504	0	100
Workload	45.980	18.930	0	100
Supportive environment^a^	76.033	17.438	0	100
Gender	0.549	0.498	0	1

^a^The number of observations for supportive environment is 43,731

**Table 2:** Frequencies for the items included into research variables

		1	2	3	4	5
*Trainee satisfaction*	Quality of teaching	21.97%	43.29%	25.18%	7.76%	1.81%
	Quality of experience	35.67%	45.72%	14.58%	3.21%	0.82%
	Quality of clinical supervision	36.75%	45.79%	14.09%	2.85%	0.52%
	Recommendation to a friend	30.82%	42.98%	19.31%	5.24%	1.65%
	Usefulness for future career	44.50%	34.58%	15.44%	4.93%	0.55%
	Acquisition of relevant competences	34.35%	46.94%	13.45%	4.30%	0.96%
	Practical experience	31.80%	43.31%	18.80%	4.96%	1.13%
*Clinical supervision*	Accessibility of senior support during on call health protection	0.28%	6.95%	6.77%	85.99%	
	Competence of the supervisor	0.91%	1.96%	3.02%	21.15%	72.97%
	Forced to cope with problems beyond competence	0.83%	5.28%	8.98%	44.06%	40.85%
	Consensus for risky procedures	0.24%	1.15%	2.06%	12.62%	83.92%
*Feedback*	Frequency of feedback	11.10%	31.66%	26.56%	26.63%	4.05%
	Feedback on personal progress	63.61%	7.47%	18.83%	6.34%	3.75%
	Feedback on performance	61.67%	6.34%	19.71%	5.36%	6.92%
*Workload*	Perceived intensity of day work	0.53%	4.07%	52.84%	32.53%	10.03%
	Perceived intensity of night work	1.58%	6.87%	45.35%	31.19%	15.01%
	Beyond contracted hours	18.74%	40.63%	13.18%	23.21%	4.24%
	Tiredness	3.98%	18.32%	20.97%	34.10%	22.63%
*Supportive environment*	Overall supportive environment	0.91%	3.65%	9.57%	48.37%	37.50%
	Fair treatment on staff	1.16%	5.17%	12.27%	52.97%	28.44%
	Respect	0.59%	2.91%	8.56%	54.58%	33.35%
	Supportive environment in confidence building	1.68%	6.37%	16.19%	48.84%	26.93%
	Superior-subordinate openness	1.50%	6.00%	21.39%	54.26%	16.85%

To identify determinants of trainee satisfaction we first ran pairwise correlation analysis between the variables included into this study. From the analysis of correlation results (Table 3), it can be found that trainee satisfaction shows a high and significant Pearson-coefficient with supportive environment (0.694), feedback (0.498), clinical supervision (0.445), whereas a less relevant but still significant correlation coefficient with workload (0.273). All other correlations achieve a significant correlation, even though their level of strength is quite low.

**Table 3:** Pairwise correlation matrix of the research variables

	Overall satisfaction	Clinical supervision	Feedback	Workload	Supportive environment	Gender
Overall satisfaction	1.0000					
Clinical supervision	0.445^a^	1.0000				
Feedback	0.498^a^	0.338^a^	1.0000			
Workload	0.273^a^	0.331^a^	0.243^a^	1.0000		
Supportive environment	0.694^a^	0.440^a^	0.419^a^	0.365^a^	1.000	
Gender	-0.027^a^	-0.054^a^	-0.059^a^	−0.002	−0.033^a^	1.0000

^a^Results are significant at the 0.01 level

Table 4 summarises OLS multiple regression results for this study. The goodness of fit of the research model is good. Indeed, models explain 34.38% (Model 1) and 55.16% (Model 2) of the variability of Trainee satisfaction. Empirical findings from the whole dataset (2012–2015) provide support to our research question, that overall trainee satisfaction is predicated upon clearly demarcated elements of their training programme, clinical practice supervision and supportive environment. In the 4 years covered by this study, UK trainees’ satisfaction in relation to their training programme was positively and significantly affected by the level of clinical supervision (β_1_ = 0.379; *p*-value < 0.001), which is the explaining variable showing the strongest effect, among those included into this study. However, results from data reported in 2015 confirms that supportive environment plays a major role in explaining trainee satisfaction (β_5_ = 0.484; *p*-value < 0.001). Feedback and workload are also positive and significant factors for predicting trainee satisfaction, though the magnitude of their effect is lower compared to the first two factors (β_2_ = 0.244; *p*-value < 0.001; β_3_ = 0.064; p-value <0.001). Overall, trainees who perceive strong clinical supervision, frequent and useful feedback meetings as well as an adequate workload are more satisfied with their training programme. The control variable, Gender, is also positively associated to the level of overall satisfaction (β_4_ = 0.109; *p*-value < 0.1), meaning that female trainees are more satisfied with their programme than their male colleagues. More recently, in the 2015 survey, this result has shown an even stronger effect (β_4_ = 0.211; *p*-value <0.05). However, this is not a modifiable factor. It can be used to explain differences in satisfaction but not to direct measures to improve satisfaction. It is worth noting that when supportive environment has been controlled also (Model 2), the effect of clinical supervision and feedback is reduced (β_1_ = 0.158; *p*-value <0.001; β_2_ = 0.131; p-value <0.001). Moreover, the workload coefficient has changed into a negative, even though the size of the effect is rather small (β_2_ = − 0.029; *p*-value <0.001).

**Table 4:** OLS regression results on Trainee satisfaction

	Model 1 Robust coefficients (SE)	Model 2 Robust coefficients (SE)
Clinical supervision	0.379 ^a^ (0.003)	0.158^a^ (0.005)
Feedback	0.244^a^ (0.001)	0.131^a^ (0.002)
Workload	0.064^a^ (0.002)	−0.029^a^ (0.003)
Gender	0.109^a^ (0.061)	0.211^a^ (0.095)
Supportive environment		0.484 ^a^ (0.003)
Constant	26.293^a^ (0.242)	22.544^a^ (0.388)
Adj-R^2^	34.38%	55.16%
F statistics	20,269.00^a^	6997.88^a^
Mean VIF	1.13	1.26
SE of regression	11.759	9.619
Observations	148,247	37,630

Robust standard errors are in brackets. The most important factor for each Model is underlined

^a^Results are statistically significant at the 0.1, 0.05 and 0.001 level

We also performed a variety of robustness tests, such as robust standard errors, robust OLS regression, and multi-co-linearity check, which show no violation of linear OLS assumptions.

Thus, within the many factors surveyed by the GMC in the 2012–2015 National Trainee Survey “Overall Satisfaction” is positively correlated with 4 key factors. These are supportive environment, workload, clinical supervision and feedback.

## Discussion

Recent reports of low morale do not apparently correlate with self-reporting of overall satisfaction with training itself by UK trainees as 83% rate their overall satisfaction as good or excellent [14]. But almost one in six (17%) gave an adverse rating (13% fair and 4% as poor or very poor), this demonstrates room for improvement and given the recent unrest dissatisfaction levels may rise. Given the importance of supportive environment it is timely to consider the factors within training programmes and employment environments that contribute most to high trainee satisfaction.

Supportive environment is unsurprisingly a key factor regarding overall satisfaction. Postgraduate medical trainees are required to fulfil broad curricula requirements over many years, demonstrate considerable numbers of competences and pass numerous assessments and summative examinations. Historically sufficient experience was acquired by working long hours, which was neither safe nor efficient. Since the introduction of the European Working Time Regulations this prolonged haphazard immersion method of medical training has rightly no longer been possible. Without massively extending training length it is therefore vital that doctors in training gain adequate experience within the hours that they work.

Workload could superficially be regarded as directly related to adequate experience. However, it is clear that these two are not directly related. Sufficient contracted hours are positively correlated with perception of workload and adequate experience. It is likely that there are core tensions of workload and adequate experience. If the activity relates to the required learning, that is the doctor’s curricular requirements, it is likely to be acceptable and positively perceived but where tasks are deemed irrelevant to that learning or the workload is such that it impedes learning opportunities then satisfaction falls. Pressure to under-report hours worked is likely to be a marker of excessive workload but also of an unsupportive learning environment.

Clinical Supervision is clearly a key hygiene factor [17]. It is likely that this relates both to the ability to provide safe care and feel supported whilst so doing but also to the ability to learn from a senior colleague. This links closely to the positive correlation to high-quality feedback. This feedback may be either positive or negative in its content but if well delivered and focussed on learning and therefore on both safe care and the individual’s learning needs it is likely to be associated with high trainee satisfaction.

Research in other employment and learning sectors identifies a number of key factors. Within the Higher Education Sector, University student perceptions of their learning environment are a strong predictor of learning outcomes. Indeed, they are a stronger predictor than the students’ prior educational achievement [22]. Similarly, in the information technology sector a positive learning culture is associated with employee satisfaction whereas a negative culture reduces satisfaction and increases “turnover intention” [10]. This suggests that further consideration should be given to the supportiveness of the environment. For example in the hospitality sector it has been shown that the organisational culture and fit of values affects employee job satisfaction [35].

## Conclusion

This paper sought to identify the main determinants of trainee satisfaction, through a validated tool. This develops our knowledge of existing metrics to evaluate the level of satisfaction of doctors in training. This allows ranking of key factors, which is in line with prior literature saying that appropriate workload [8, 9], good supervision of practice [39, 23] and the receipt of good quality feedback [28] are crucial for high trainee satisfaction and in contrast with some scholars focusing on the skills and knowledge by trainees in their programme only [32]. Clinical supervision is the most important factor among this triad. However, the support junior doctors receive from the environment where they train is considered as the most important predictor of trainee satisfaction.

Despite the cessation of industrial action by Junior Doctors in the UK it is clear that more must be done to rebuild relations. It is vital that doctors in training are supported to be the best doctors that they can be and valued in such a manner that best encourages them to remain in the service of the NHS.

The evidence shows that supportive environment is the most important factor contributing to trainee satisfaction. Therefore, policy-makers should primarily focus on ensuring a supportive culture within healthcare and education providers. This might involve inclusion in quality assurance processes and performance outcomes metrics, such as the culture of care barometer [27], testing the environments with surveys that encompass other health care workers (e.g. the annual NHS staff survey) and including environment and culture in organisational development plans.

Regulators and funding bodies should recognise the importance of sufficient and capable clinical supervision. Prioritising selection, training (including regarding feedback), assessment and appropriate remuneration of clinical supervisors. Furthermore, employers should ensure that sufficient protected time for the provision of safe, high quality clinical supervision is included in clinical educators’ job plans.

### Further research

Whilst this work has focussed on the evidence of factors relating to Junior Doctor satisfaction we suggest that their feedback could also be linked to the economic costs of their training as in the post-industrial action discussions these doctors have complained about increasing costs of required courses and of mandatory examinations [16]. It may also be related to the quality of service provision as many of the clinical learning environments within which these doctors train have been rated poorly for the quality of service [6]. Although this is a comprehensive and rigorous study, it is based in a specific national context. Thus, additional research effort could be addressed to considering the impact of cost, service quality and the factors that contribute to junior doctor satisfaction in other nations. This might help in corroborating preliminary findings.

Also, more qualitative analysis on the determinants of junior doctor satisfaction should be addressed, in order to better understand the basis of junior doctor morale and their needs.

Our findings on workload do not show either a direct or an indirect correlation to trainee satisfaction. We, therefore, recommend further research in this area. Consideration should be given to whether there is a nonlinear relationship between workload and trainee satisfaction.

### Practical implications

Doctors and their educators should review their priorities to ensure balance of the provision of clinical service and clinical supervision, recognising that clinical supervision positively contribute to trainee satisfaction and probably to higher quality patients care. Employers should ensure that job descriptions, contracts and weekly timetables (job plans) should value and reward professional activities such as clinical supervision in addition to direct patients contact.

## Additional files

Additional file 1: Questionnaire. This file provides information regarding the questions included into the survey related to this study. http://www.gmc-uk.org/2017_BN3_Annex_A_pdf.pdf_71025099.pdf. (PDF 141 kb)

Additional file 2: Factor analysis of the research variables [25]. (DOCX 115 kb)

## Abbreviations

GMC: General Medical Council
PMETB: Postgraduate Medical Education and Training Board
