# Supplementary material for: Satisfaction of doctors with their training: evidence from UK
Source: BMC Health Serv Res. 2017 Dec 29;17:851. doi: 10.1186/s12913-017-2792-0 (PMC5747190; doi:10.1186/s12913-017-2792-0)
Supplement: Supplementary file 1 — Questionnaire. This file provides information regarding the questions included into the survey related to this study. http://www.gmc-uk.org/2017_BN3_Annex_A_pdf.pdf_71025099.pdf. (PDF 141 kb) [file 12913_2017_2792_MOESM1_ESM.pdf]

Briefing note 3 – Annex A

Generic and demographic final questionnaire

The following pages contain the final questionnaire for generic (including Public Health trainees) and demographic questions for the 2015 National Training Survey.

Each row represents an individual question (or the text on the page).

The second column shows the question code.

Branching is shown in the final column. Alternative wording for Public Health trainees is shown in grey text.

Question number	Question	Answers	Area	Branching
	The survey will begin on the next page and is made up of the following sections:<br>Opening section: A few questions about your working arrangements.<br>Estimated time for completion: 1 minute.<br>Main section: Questions about your post.<br>Estimated time for completion: 10 minutes.<br>Academic section: If you are an academic trainee there are some questions about your experiences as an academic.<br>Estimated time for completion: 5 minutes.<br>Programme section: Questions about your programme (Foundation, Core Training or Specialty Training). A small number of programmes have no questions in this section.<br>Estimated time for completion: Between 1 and 5 minutes<br>Closing section<br>A few demographic questions and an opportunity for you to raise patient safety concerns.<br>Estimated time for completion: 2 minutes.<br>For most people the total time for completion will be between 15 and 20 minutes.			
OPENQ11	Are you working less than full time?	Yes | No	Demographic	

Question number	Question	Answers	Area	Branching
OPENQ09	How many hours per week are you contracted to work?<br><br>In some weeks you may work more than 48 hours. However, please give your average over your post.	20 or less|21-30|31-40|41-48|49-56|More than 56	Demographic	
OPENQ10	On average, how many hours per week do you ACTUALLY work in this post?<br><br>In some weeks you may work more than 48 hours. However, please give your average over your post.	20 or less|21-30|31-40|41-48|49-56|More than 56	Demographic	
**Thank you for completing the questions about your working arrangements. Click next to move to the next section.**				
Questions about your post. Estimated time for completion: 10 minutes				
GENHQ02	Did you get all the information you needed about your workplace when you started working in this post?	Yes | No | Not sure | Not Applicable	Induction	
GENHQ03	Did someone explain your role and responsibilities in your unit or department at the start of this post?	Yes | No | Not sure | Not Applicable	Induction	
GENHQ04	How would you rate the quality of induction in this post? (This refers to your induction to the organisation in which you worked.)	Excellent | Good | Fair | Poor | Very poor	Induction	
GENHQ05	Did you have a designated educational supervisor (the person responsible for your appraisal) in this post?	Yes | No | Not sure	Educational supervision	
GENHQ06	Did you sit down with your educational supervisor and discuss your educational objectives for this post?	Yes | No | Not sure | Not Applicable	Induction	
GENHQ07	In this post did you have a training/learning agreement with your educational supervisor, setting out your respective responsibilities?	Yes | No | Not sure | Not Applicable	Educational supervision	
GENHQ91	Please state whether you agree or disagree with the following statements about your post:<br><br>My educational supervisor would be helpful if I were to have any concerns that affect my training	Strongly agree | Agree | Neither agree nor disagree | Disagree | Strongly disagree | Not applicable	Educational supervision new	
GENHQ92	Please state whether you agree or disagree with the following statements about your post:<br><br>My educational supervisor is accessible should I need to contact them	Strongly agree | Agree | Neither agree nor disagree | Disagree | Strongly disagree | Not applicable	Educational supervision new	

Question number	Question	Answers	Area	Branching
GENHQ93	Please state whether you agree or disagree with the following statements about your post:<br><br>The level of input from my educational supervisor is appropriate for my training needs	Strongly agree | Agree | Neither agree nor disagree | Disagree | Strongly disagree | Not applicable	Educational supervision new	
GENHQ08	In this post did you use a learning portfolio?	Yes | No | Not sure | Not Applicable	Educational supervision	
GENHQ08	In this post did you use a learning portfolio/evidence folder? (Pharmaceutical Medicine only)	Yes | No | Not sure | Not Applicable	Educational supervision	
GENHQ09	In this post were you told who to talk to in confidence if you had concerns, personal or educational?	Yes | No | Not sure | Not Applicable	Educational supervision	
GENHQ94	Please state whether you agree or disagree with the following statement about your post:<br><br>My educational supervisor's input into my e-portfolio is appropriate for my training needs	Strongly agree | Agree | Neither agree nor disagree | Disagree | Strongly disagree | Not applicable	Educational supervision new	
GENHQ10	How would you rate the quality of teaching (informal and bedside teaching as well as formal and organised sessions) in this post?	Excellent | Good | Fair | Poor | Very poor	Overall satisfaction	
GENPQ10	How would you rate the quality of teaching (informal as well as formal and organised sessions) in this post? (Public Health only)	Excellent | Good | Fair | Poor | Very poor	Overall satisfaction	
GENPQ10	How would you rate the quality of teaching/coaching (informal as well as formal and organised sessions) in this post? (Pharmaceutical Medicine only)	Excellent | Good | Fair | Poor | Very poor	Overall satisfaction	
GENHQ15	In this post who provided the local/departmental teaching?	Other trainees without senior supervision | Other trainees with senior supervision | A mixture of both trainees and seniors | Senior doctors | Other e.g. Specialist Nurse	Local teaching	
GENPQ15	In this post who provided the local/departmental teaching? (Public Health only)	Other trainees without senior supervision | Other trainees with senior supervision | A mixture of both trainees and seniors | Senior doctors | Other e.g. specialist nurse	Local teaching	
GENHQ11	How would you rate the quality of this local/departmental teaching for this post?	Excellent | Good | Fair | Poor | Very poor	Local teaching	

Question number	Question	Answers	Area	Branching
GENHQ12	For how many hours per week was the local/departmental basis specialty-specific teaching provided in this post?	Less than 1 hr | 1-2 hrs | 2-4 hrs | 5-8 hrs | More than 8 hrs	Local teaching	
GENHQ13	When attending these local/departmental sessions, in this post, how often did you have to leave a teaching session to answer a clinical call?	Never, it was protected time | Never, but it was not specifically protected time | Some sessions | Once every session | Multiple times each session	Local teaching	
GENHQ14	When attending these local/departmental sessions in this post who covered your service work? (Please tick all the options that apply).	Not covered | Designated trainee who would otherwise attend teaching | Designated trainee who would not attend teaching anyway | Designated senior doctor | Nurse specialist | Other	Local teaching	
GENHQ16	In this post, was specialty-specific teaching provided on a deanery/regional/school wide basis?	Yes - all of it | Yes - most of it | No	Regional teaching	"Yes - all of it" and "Yes - most of it" branch to GENHQ17, "No" branches to GENHQ20
GENHQ17	In this post how frequently was this deanery/regional/school specialty-specific teaching provided?	Weekly | Fortnightly | Monthly | Bi-monthly | Less frequently	Regional teaching	
GENHQ18	Were you able to attend these whilst in this post?	Yes, every time | Yes, most of the time | Yes, some of the time | No | Not applicable - none have taken place yet	Regional teaching	
GENHQ19	How would you rate the quality of this deanery/regional/school specialty-specific teaching for this post?	Excellent | Good | Fair | Poor | Very poor	Regional teaching	
GENHQ20	Overall, how would you rate the educational resources available to you in this post?	Very good | Good | Neither good nor poor | Poor | Very poor | Not applicable	Access to educational resources	
GENHQ21	How good or poor was access to each of the following in your post?<br><br>Library	Very good | Good | Neither good nor poor | Poor | Very poor | Not applicable	Access to educational resources	
GENHQ22	How good or poor was access to each of the following in your post?<br><br>Online journals	Very good | Good | Neither good nor poor | Poor | Very poor | Not applicable	Access to educational resources	
GENHQ23	How good or poor was access to each of the following in your post?<br><br>E-learning resources	Very good | Good | Neither good nor poor | Poor | Very poor | Not applicable	Access to educational resources	

Question number	Question	Answers	Area	Branching
GENHQ24	How good or poor was access to each of the following in your post?<br><br>Internet access	Very good | Good | Neither good nor poor | Poor | Very poor | Not applicable	Access to educational resources	
GENHQ25	How good or poor was access to each of the following in your post?<br><br>Space for private study	Very good | Good | Neither good nor poor | Poor | Very poor | Not applicable	Access to educational resources	
GENHQ26	How good or poor was access to each of the following in your post?<br><br>Equipped rooms for group teaching	Very good | Good | Neither good nor poor | Poor | Very poor | Not applicable	Access to educational resources	
GENHQ27	How good or poor was access to each of the following in your post?<br><br>Simulation facilities	Very good | Good | Neither good nor poor | Poor | Very poor | Not applicable	Access to educational resources	
GENHQ50	In this post how would you rate the encouragement you had to take study leave?	Excellent | Very good | Good | Neither good nor poor | Poor | Very poor	Study leave	
GENHQ51	In this post were you able to access funds to cover the cost of all courses that were recommended for you to complete?	Yes | No | Not Applicable	Study leave	
GENHQ52	Were any days subtracted from your study leave allowance for compulsory training in this post (in or out of hospital)?	Yes | No |Don’t know	Study leave	
GENPQ52	Were any days subtracted from your study leave allowance for compulsory training in this post? (Public Health and Pharmaceutical Medicine only)	Yes | No | Don't know	Study leave	
GENHQ53	In this post did you have difficulty obtaining study leave for any of the following reasons? (Please tick all the options that apply).	No difficulty | Yes - fixed leave pattern | Yes - other difficulties due to local rota policies | Yes - failure to find prospective cover | Yes - active discouragement from seniors | Yes - leave refused as reason deemed educationally inappropriate/unhelpful | Yes - administrative difficulties	Study leave	
GENHQ28	How often did you have informal feedback from a supervisor/public health consultant/senior colleague on how you were doing in this post?	Daily | Weekly | Monthly | Rarely | Never	Feedback	
GENHQ28	How often did you have informal feedback from a supervisor/public health consultant/senior colleague on how you were doing in this post? (Public Health only)	Daily | Weekly | Monthly | Rarely | Never	Feedback	

Question number	Question	Answers	Area	Branching
GENHQ28	How often did you have informal feedback from a supervisor/pharmaceutical medicine consultant/senior colleague on how you were doing in this post? (Pharmaceutical Medicine only)	Daily | Weekly | Monthly | Rarely | Never	Feedback	
GENHQ29	Did you have a formal meeting with your supervisor to talk about your progress in this post?	Yes, and it was useful | Yes, but it wasn't useful | No, but this will happen | No, but it wasn't necessary | No, but I would like to	Feedback	
GENHQ30	Did you have a formal assessment of your performance in the workplace in this post?	Yes, and it was useful | Yes, but it wasn't useful | No, but this will happen | No, but it wasn't necessary | No, but I would like to	Feedback	
GENHQ95	Please state whether you agree or disagree with the following statements about your post:<br><br>Handover arrangements in this post BETWEEN SHIFTS ensure continuity of care for patients	Strongly agree | Agree | Neither agree nor disagree | Disagree | Strongly disagree | Not applicable	Handover new	
GENHQ96	Please state whether you agree or disagree with the following statements about your post:<br><br>Handover of patients BETWEEN DEPARTMENTS in this post ensures continuity of care	Strongly agree | Agree | Neither agree nor disagree | Disagree | Strongly disagree | Not applicable	Handover new	
GENHQ97	Please state whether you agree or disagree with the following statements about your post:<br><br>Appropriate members of the multidisciplinary team are included in handover	Strongly agree | Agree | Neither agree nor disagree | Disagree | Strongly disagree | Not applicable	Handover new	
GENHQ31	In this post, which of the following best describes handover arrangements BEFORE night duty?	Not applicable | An organised meeting of doctors and nurses | An organised meeting of doctors | Phone or email communication | Informal | No arrangements	Handover	
GENHQ32	In this post, which of the following best describes handover arrangements AFTER night duty?	Not applicable | An organised meeting of doctors and nurses | An organised meeting of doctors | Phone or email communication | Informal | No arrangements	Handover	
GENHQ33	How would you rate the intensity of your work, by day in this post?	Very light | Light | About right | Heavy | Very heavy	Workload	
GENHQ34	How would you rate the intensity of your work, by night in this post?	Not applicable | Very light | Light | About right | Heavy | Very heavy	Workload	

Question number	Question	Answers	Area	Branching
GENHQ98	Please state whether you agree or disagree with the following statements about your post:<br><br>The working PATTERN in this post allows me enough sleep so that I can safely manage my patients	Strongly agree | Agree | Neither agree nor disagree | Disagree | Strongly disagree | Not applicable	Workload new	
GENHQ99	Please state whether you agree or disagree with the following statements about your post:<br><br>The work INTENSITY in this post allows me to safely manage my patients	Strongly agree | Agree | Neither agree nor disagree | Disagree | Strongly disagree | Not applicable	Workload new	
GENHQ35	In this post, how often have you worked beyond your rostered hours? (excl. Pharmaceutical Medicine)	Daily | Weekly | Monthly | Rarely | Never	Workload	
GENHQ35	In this post, how often have you worked beyond your contracted hours? (Pharmaceutical Medicine only)	Daily | Weekly | Monthly | Rarely | Never	Workload	
GENHQ61	In this post how often, if at all, have you been asked to or felt pressured to submit a record of hours worked that were less than the hours you actually worked?	Never | Once | More than once	Demographic	
GENHQ36	In this post, how often did your working pattern leave you feeling short of sleep when at work?	Daily | Weekly | Monthly | Rarely | Never	Workload	
GENHQ37	In this post did you always know who your available senior support was during on call health protection?	Yes and they were accessible | Yes, but they were not easy to access | No, but there was usually someone I could contact | No, there was no one I could contact | Not applicable	Clinical supervision	
GENPQ37	In this post did you always know who your available senior support was during on call health protection? (Public Health only)	Yes and they were accessible | Yes, but they were not easy to access | No, but there was usually someone I could contact | No, there was no one I could contact | Not applicable	Clinical supervision	
GENHQ38	In this post how often, if ever, were you supervised by someone who you felt wasn't competent to do so?	Daily | Weekly | Monthly | Rarely | Never	Clinical supervision	
GENHQ39	In this post how often did you feel forced to cope with clinical problems beyond your competence or experience?	Daily | Weekly | Monthly | Rarely | Never	Clinical supervision	
GENPQ39	In this post how often did you feel forced to cope with problems beyond your competence or experience? (Public Health only)	Daily | Weekly | Monthly | Rarely | Never	Clinical supervision	

Question number	Question	Answers	Area	Branching
GENHQ40	In this post how often have you been expected to obtain consent for procedures where you feel you do not understand the proposed interventions and its risks?	Daily | Weekly | Monthly | Rarely | Never | Not applicable	Clinical supervision	
GENHQ70	In this post have you worked out of hours (this includes night shifts and weekends)?	Yes | No | Not applicable	Clinical supervision out of hours	'Yes' branches to GENHQ71
GENHQ71	In this post, OUT OF HOURS, did you always know who was providing your clinical supervision when you were working?	Yes and they were accessible | Yes, but they were not easy to access | No, but there was usually someone I could contact | No, there was no one I could contact | Not applicable	Clinical supervision out of hours	
GENHQ72	In this post, OUT OF HOURS, how often, if ever, were you clinically supervised by someone who you felt wasn't competent to do so?	Daily | Weekly | Monthly | Rarely | Never	Clinical supervision out of hours	
GENHQ73	In this post, OUT OF HOURS, how often did you feel forced to cope with clinical problems beyond your competence or experience?	Daily | Weekly | Monthly | Rarely | Never	Clinical supervision out of hours	
GENHQ74	In this post, OUT OF HOURS, how often have you been expected to obtain consent for procedures where you feel you do not understand the proposed interventions and its risks?	Daily | Weekly | Monthly | Rarely | Never | Not applicable	Clinical supervision out of hours	
GENHQ75	How would you rate the quality of clinical supervision, OUT OF HOURS, in this post?	Excellent | Good | Fair | Poor | Very poor	Clinical supervision out of hours	
GENHQ78	Please state whether you agree or disagree with the following statement about your post:<br><br>In general, the working environment is a supportive one.	Strongly agree | Agree | Neither agree nor disagree | Disagree | Strongly disagree	Supportive environment	
GENHQ79	Please state whether you agree or disagree with the following statement about your post:<br><br>Staff, including doctors in training, are treated fairly.	Strongly agree | Agree | Neither agree nor disagree | Disagree | Strongly disagree	Supportive environment	
GENHQ80	Please state whether you agree or disagree with the following statement about your post:<br><br>Staff, including doctors in training, treat each other with respect.	Strongly agree | Agree | Neither agree nor disagree | Disagree | Strongly disagree	Supportive environment	

Question number	Question	Answers	Area	Branching
GENHQ81	Please state whether you agree or disagree with the following statement about your post:<br><br>The working environment is one which helps build the confidence of doctors in training.	Strongly agree | Agree | Neither agree nor disagree | Disagree | Strongly disagree	Supportive environment	
GENHQ82	Please state whether you agree or disagree with the following statement about your post:<br><br>If I were to disagree with senior colleagues, they would be open to my opinion.	Strongly agree | Agree | Neither agree nor disagree | Disagree | Strongly disagree	Supportive environment	
GENHQ47	How often, if at all, have you been the victim of bullying and harassment in this post?	Every day | At least once per week | At least once per fortnight | At least once per month | Less often than once per month | Never | Prefer not to answer	Undermining	
GENHQ48	How often, if at all, have you witnessed someone else being the victim of bullying and harassment in this post?	Every day | At least once per week | At least once per fortnight | At least once per month | Less often than once per month | Never | Prefer not to answer	Undermining	
GENHQ49	In this post, how often if at all, have you experienced behaviour from a consultant/GP that undermined your professional confidence and/or self esteem?	Every day | At least once per week | At least once per fortnight | At least once per month | Less often than once per month | Never | Prefer not to answer	Undermining	

Question number	Question	Answers	Area	Branching
	**Raising written bullying/undermining concerns about your training post**<br><br>**What is the process?**<br><br>1. We require that all concerns will be investigated by your deanery/LETB (LETB is the name for deaneries in England).<br><br>2. To investigate your comment, we will share the following with your deanery/LETB:<br>• Your verbatim comment<br>• Your training site<br>• Your post specialty<br>• Your programme specialty<br>• Your training level<br><br>3. The deanery/LETB will liaise directly with the organisation/trust you are working for (your employer), in order to undertake a thorough investigation, as appropriate.<br><br>4. We check each deanery/LETB response, to ensure that we are satisfied with the outcome.		Bullying and undermining	

<table>
<tr><th>Question number</th><th>Question</th><th>Answers</th><th>Area</th><th>Branching</th></tr>
<tr><td></td><td colspan="2">

Am I guaranteed anonymity?

No.

Your individual answers to the multiple choice questions in the survey will always remain confidential.

Concerns about bullying/undermining that you raise within the survey will also be treated as confidential, and will not be made public by the GMC or shared outside the GMC’s Education Directorate. However, because of the importance of ensuring a safe training environment, this is subject to three exceptions.

Firstly, as explained above we will share your verbatim comment and other information about you with your deanery/LETB so that they can investigate your concern.

We will not routinely share your identity when we share your concern. However, in some cases, the deanery/LETB may ask who you are so they can ask you for further details about your concern. In this case, we will share your identity. This is because of the importance of addressing any issues that can create an unsafe training environment. We will inform you before we do this.

Secondly, if the concern you raise about bullying/undermining becomes relevant to a fitness to practise investigation then we will share your comment with the Fitness to Practise Directorate. We will inform you before we do this. This could include circumstances where fitness to practise proceedings are taken against a doctor, where there are grounds to believe that doctor has raised a concern that is not honest or made in good faith.

We will share comments with appropriate regulatory bodies where there is a legitimate need to do so

In all of the above situations, we expect your full co-operation with the process. We value your openness and transparency and we will support you provided that you act honestly and in good faith.

Raising concerns about a doctor's fitness to practise

It is not appropriate to raise concerns about a doctor’s fitness to practise here in the national training survey. Your comment in the survey is not a fitness to practise referral. If your concern is about the fitness to practise of a doctor, please see the guidance on raising concerns on our website, where you can also find details of our confidential helpline.

</td><td></td><td></td></tr>
<tr><td>GENHQ84</td><td>Do you wish to raise a bullying or undermining concern here?</td><td>Yes | No</td><td>Bullying and undermining</td><td>“Yes” branches to GENHQ85, “No” branches to GENHQ100</td></tr>
</table>

Question number	Question	Answers	Area	Branching
GENHQ85	**Your bullying or undermining concern**<br><br>Please use the text box below.<br><br>Your comment will be taken seriously and investigated. This means that it is your responsibility to:<br>• write factually and accurately about your own experience, not hearsay<br>• describe specific incidents<br>• describe specific behaviours<br><br>Please note there is a limit of 2,000 characters within the box. If you exceed the limit, you will encounter an error message.<br><br>Once finished, please use the categorisation questions below.	Free text	Bullying and undermining	
GENHQ87	Please specify who has been doing the undermining/bullying described in your concern (please select all that apply)	Consultant/GP (within my post) | Consultant/GP (outside my post) | Nurse/midwife | Other doctor | Other trainee | Management | Patient/relative | Other (please specify)	Bullying and undermining	
GENHQ88	If you selected 'other' please provide a description.	Free text	Bullying and undermining	
GENHQ89	Which behaviour types describe your concern? (Please select all that apply)	Belittling or humiliation | Threatening or insulting behaviour | Deliberately preventing access to training | Bullying relating to a protected characteristic | Other (please specify)	Bullying and undermining	
GENHQ100	Please state whether you agree or disagree with the following statements about your post:<br><br>I have been made aware of how to report patient safety incidents and near misses	Strongly agree | Agree | Neither agree nor disagree | Disagree | Strongly disagree	Reporting systems	
GENHQ101	Please state whether you agree or disagree with the following statements about your post:<br><br>There is a culture of proactively reporting concerns	Strongly agree | Agree | Neither agree nor disagree | Disagree | Strongly disagree	Reporting systems	

Question number	Question	Answers	Area	Branching
GENHQ102	Please state whether you agree or disagree with the following statements about your post:<br><br>There is a culture of learning lessons from concerns raised	Strongly agree | Agree | Neither agree nor disagree | Disagree | Strongly disagree	Reporting systems	
GENHQ103	Please state whether you agree or disagree with the following statements about your post:<br><br>I am confident that concerns are effectively dealt with	Strongly agree | Agree | Neither agree nor disagree | Disagree | Strongly disagree | N/A (not aware of any concerns being raised)	Reporting systems	
GENHQ104	Please state whether you agree or disagree with the following statements about your post:<br><br>When concerns are raised, the subsequent actions are fed back appropriately	Strongly agree | Agree | Neither agree nor disagree | Disagree | Strongly disagree | N/A (not aware of any concerns being raised)	Reporting systems	
GENHQ54	How would you rate the quality of clinical supervision in this post? (excl. Public Health and Pharmaceutical Medicine)	Excellent | Good | Fair | Poor | Very poor	Overall satisfaction and Clinical supervision	
GENPQ54	How would you rate the quality of supervision in this post? (Public Health and Pharmaceutical Medicine only)	Excellent | Good | Fair | Poor | Very poor	Overall satisfaction and Clinical supervision	
GENHQ55	How would you rate the quality of experience in this post?	Excellent | Good | Fair | Poor | Very poor	Overall satisfaction	
GENHQ56	How would you rate the practical experience you were receiving in this post?	Excellent | Good | Fair | Poor | Very poor	Adequate experience	
GENHQ57	How confident are you that this post will help you acquire the competencies you needed at that particular stage of your training?	Very confident | Fairly confident | Neutral | Not very confident | Not at all confident	Adequate experience	
GENHQ58	How would you describe this post to a friend who was thinking of applying for it?	Excellent | Good | Fair | Poor | Very poor	Overall satisfaction	
GENHQ59	How useful do you feel this post will be for your future career?	Very useful | Useful | Fairly useful | Not very useful | Useless	Overall satisfaction	

Question number	Question	Answers	Area	Branching
GENHQ76	Which, if any, of the following academic trainee roles do you currently hold?<br><br>Please note - an academic traineeship occurs when a trainee is undertaking formal academic training alongside their clinical training or has taken time out of clinical training to undertake academic training.	I am not an academic trainee | Academic Foundation Trainee (AFT) | NIHR Clinical Lecturer - England (NIHR funding) (CL) | Clinical Lecturer - England (other funding), Wales (CL) | Clinical Lecturer - Scotland | Academic Clinical Lecturer - Northern Ireland (ACL) | NIHR Academic Clinical Fellow - England (NIHR funding) (ACF) | Academic Clinical Fellow - England (other funding), Wales, Northern Ireland (ACF) | Clinical Research Fellow - Scotland | Clinical Teaching Fellow - Scotland | Other academic role	Academic	"I am not an academic trainee" branches to programme specific questions, if applicable, all other answers branch to GENHQ77.
GENHQ77	Have you used your protected research time to undertake research in the last 12 months?	Yes | No	Academic	"Yes" branches to academic questionnaire, "No" branches to programme specific questions.
	**Thank you for completing the questions about your post. Click next to move to the next section.**			
Closing questions. Estimated completion time: 2 minutes				
CLSGQ02	We would appreciate your help in completing the following Equality and Diversity Monitoring questions. Any answers you give will be used to help us to fulfil our statutory duties and our responsibilities under the Equality Act 2010. For example, your information will help us regulate medical education and training and ensure progression through GMC approved training programmes is fair and free from discrimination. We will aggregate and anonymise any information we publish so that your answers cannot be identified.<br><br>What is your ethnic group?	White - English/Welsh/Scottish/Northern Irish/British | White - Irish | White - Gypsy or Irish Traveller | White - Any other White background (please write in) | Mixed/Multiple ethnic groups - White and Black Caribbean | Mixed/Multiple ethnic groups - White and Black African | Mixed/Multiple ethnic groups - White and Asian | Mixed/Multiple ethnic groups - Any other Mixed/multiple ethnic background (please write in) | Asian/Asian British - Indian | Asian/Asian British - Pakistani | Asian/Asian British - Bangladeshi | Asian/Asian British - Chinese | Asian/Asian British - Any other Asian background (please write in) | Black/African/Caribbean/Black British - African | Black/African/Caribbean/Black British - Caribbean | Black/African/Caribbean/Black British - Any other Black/African/Caribbean background (please write in) | Other ethnic group - Arab | Other ethnic group - Any other ethnic group (please write in) | Prefer not to say	Demographic	"Other ethnic group – Any other ethnic group (Please write in)" branch to CLSGQ03, all others branch to CLSGQ50?

Question number	Question	Answers	Area	Branching
CLSGQ03	Ethnicity description. If you selected 'other' please provide a description of your ethnicity.	Free text	Demographic	
CLSGQ50	Are you disabled?	Yes | No | Don't know | Prefer not to say	Demographic	
CLSGQ51	Are your day-to-day activities limited because of a health problem or disability which has lasted, or is expected to last, at least 12 months?	Yes, limited a lot | Yes, limited a little | No | Prefer not to say	Demographic	“Yes, limited a lot” and “Yes limited a little” branch to CLSGQ05, “No” and “Prefer not to say” branch to CLSGQ25
CLSGQ05	In this post, do/did you require adjustment(s) to be made so you can carry out your work?	Yes | No	Demographic	“Yes” branches to CLSGQ06, “No” branches to CLSGQ25
CLSGQ06	Have any adjustment(s) been made so you can carry out your work in this post?	Yes - all adjustments have been made | Yes – some adjustments have been made but not all of them | No – the adjustments I need have not been made	Demographic	“Yes - all adjustments have been made | Yes – some adjustments have been made but not all of them” branches to CLSGQ07?
CLSGQ07	Please insert any comments you have about the adjustments you require.	Free text	Demographic	

Question number	Question	Answers	Area	Branching
CLSGQ75	**Patient Safety**<br><br>You now have an opportunity to raise any patient safety concerns about your post.<br><br>**All doctors have a duty to act when they believe patient safety is at risk, or when a patient's dignity or comfort is being compromised. This includes raising concerns through local channels when they arise.**<br><br>**The organisation where you are currently working may be the most appropriate and effective place for you to raise the concern and this should be your first consideration.**<br><br>**What is the process?**<br><br>1. We require that all concerns raised in your response to this question should be investigated by your deanery/LETB (LETB is the name for deaneries in England).<br><br>2. To investigate your comment, we will share the following with your deanery/LETB:<br>• Your verbatim comment<br>• Your training site<br>• Your post specialty<br>• Your programme specialty<br>• Your training level<br><br>3. The deanery/LETB will liaise directly with the organisation/trust you are working for, in order to undertake a thorough investigation, as appropriate.<br><br>4. We check each deanery/LETB response, to ensure that we are satisfied with the outcome.		Patient safety	

Question number	Question	Answers	Area	Branching
CLSGQ88	**Am I guaranteed anonymity?**<br><br>No.<br><br>Your individual answers to the multiple choice questions in the survey will always remain confidential.<br><br>Concerns about patient safety that you raise within the survey will also be treated as confidential, and will not be made public by the GMC or shared outside the GMC's Education Directorate. However, because patient safety must come first, this is subject to three exceptions.<br><br>1. Firstly, as explained above we will share your verbatim comment and other information about you with your deanery/LETB so that they can investigate your concern.<br><br>We will not routinely share your identity when we share your concern. However, in some cases, the deanery/LETB may ask who you are so they can ask you for further details about your concern. In this case, we will share your identity. This is because our first priority must be the care of patients. We will inform you before we do this.<br><br>2. Secondly, if the concern you raise about patient safety becomes relevant to a fitness to practise investigation then we will share your comment with the Fitness to Practise Directorate. We will inform you before we do this. This could include circumstances where fitness to practise proceedings are taken against a doctor, where there are grounds to believe that doctor has raised a concern that is not honest or made in good faith.<br><br>3. We will share comments with appropriate regulatory bodies where there is a legitimate need to do so.<br><br>In all of the above situations, we expect your full co-operation with the process. We value your openness and transparency and we will support you provided that you act honestly and in good faith.			
CLSGQ90	**Raising concerns about a doctor's fitness to practise**<br><br>It is not appropriate to raise concerns about a doctor's fitness to practise here in the national training survey. Your comment in the survey is not a fitness to practise referral. If your concern is about the fitness to practise of a doctor, please see the guidance on raising concerns on our website, where you can also find details of our confidential helpline.			

<table>
<tr><th>Question number</th><th>Question</th><th>Answers</th><th>Area</th><th>Branching</th></tr>
<tr><td>CLSGQ60</td><td>In this post, have you had any concerns about patient safety?</td><td>No | Yes, but they are already being addressed, or have been resolved | Yes, and they have not yet been addressed</td><td>Patient safety</td><td>“No” and “Yes, but they are already being addressed, or have been resolved” branch to end of survey, “Yes, and they have not yet been addressed” branches to additional guidance on page 45.</td></tr>
<tr><td>CLSG78</td><td colspan="2">What to include in your comment

Please include in your comment (as appropriate):
• a clear description of the incident or process giving rise to the risk, including location (for example: ward)
• use accurate and factual examples relating to your personal experience, not hearsay
• avoid commenting on wider general service issues which do not relate to a specific incident
• if appropriate, suggest the improvements you believe would secure the safety of patients

When finished, please answer the questions below.

Please note there is a limit of 2,000 characters within the box. If you exceed the limit, you will encounter an error message.

[free text]</td><td>Patient safety</td><td></td></tr>
<tr><td>CLSGQ63</td><td>If you work across multiple sites please tell us the Trust and/or Site where the concern applies.</td><td>Free text</td><td>Patient safety</td><td></td></tr>
<tr><td>CLSGQ65</td><td>When did you first become concerned about patient safety in your post?</td><td>Within the last month | Over a month ago but less than 3 months ago | Over three months ago</td><td>Patient safety</td><td></td></tr>
<tr><td>CLSGQ85</td><td>As far as you are aware, has this patient safety concern been reported (for example, to your employer or another body)?</td><td>Yes | No | Don’t know</td><td>Patient safety</td><td>‘Yes’ branches to next question</td></tr>
</table>

Question number	Question	Answers	Area	Branching
CLSGQ86	Who was the patient safety concern reported to?	My employer | My deanery/LETB | GMC | Another body | Don't know	Patient safety	
CLSG84	**Thank you for raising a patient safety concern**<br><br>The safety of patients is our first concern and we will now work with your deanery/local education and training board (LETB) and post provider to review the information you have provided and investigate the problem where appropriate.<br><br>**Next steps**<br>• We will share your verbatim comment with your deanery/LETB within five working days.<br>• We will not automatically share your identity, but we will tell them your training site, post specialty and training level to help locate the concern<br>• The deanery/LETB will liaise directly with the organisation you are training in, in order to undertake a thorough investigation, if one is necessary.<br>• In some cases we will need to tell your deanery/LETB and your placement provider who you are to enable a thorough investigation of the problem. If we do this, we will let you know by email.<br><br>You can read more about what happens to concerns raised in the survey on our website and for more information about confidentiality please read our data protection notice.		Patient safety	
CLSGQ87	We keep a list of doctors who are interested in helping us develop improvements to the survey. From time to time we contact people on this list and invite them to comment on our work.<br>This might be at a meeting or an event, in a phone call or to simply read something we send in an email.<br>There is of course no obligation for people on the list to participate, and you can be removed from the list upon request. If you're interested in helping us develop the survey, and you want to be on our list of contacts for this purpose, please select the appropriate response below.	Yes, please add me to the list | No thanks		

Thank you for completing the final questions on the survey. Click next to move to the next screen which will show your completion code.
