# Supplementary material for: Satisfaction of doctors with their training: evidence from UK
Source: BMC Health Serv Res. 2017 Dec 29;17:851. doi: 10.1186/s12913-017-2792-0 (PMC5747190; doi:10.1186/s12913-017-2792-0)
Supplement: Supplementary file 2 — Factor analysis of the research variables [25]. (DOCX 115 kb) [file 12913_2017_2792_MOESM2_ESM.docx]

Appendix A – Factor analysis of the research variables

| Research variable | Item | Factor loading | Eigenvalue | Cronbach’s alpha |
| --- | --- | --- | --- | --- |
| *Overall satisfaction* | Quality of teaching  Quality of experience  Quality of clinical supervision  Recommendation of the post to a friend  Usefulness of the post for future career | 0.493  0.827  0.568  0.793  0.832 | 9.140 | 0.892 |
| *Adequate experience* | Acquisition of the needed competences  Practical experience received in a post | 0.841  0.824 |  | 0.812 |
| *Clinical supervision* | Accessibility of senior support when requested  Competence of the supervisor  Cope with problems beyond competence  Consent for high risk procedures | 0.354  0.639  0.688  0.697 | 1.213 | 0.665 |
| *Feedback* | Frequency of feedback  Personal progress meeting  Performance assessment | 0.487  0.798  0.813 | 1.480 | 0.669 |
| *Workload* | Perceived intensity of day work  Perceived intensity of night work  Beyond contracted hours  Tiredness | 0.768  0.654  0.642  0.624 | 1.576 | .680 |
| *Supportive environment* | Overall supportive environment  Fair treatment on staff  Respect  Supportive environment in confidence building  Openness | 0.741  0.774  0.807  0.747  0.681 | 2.073 | 0.898 |

Research models

$Trainee satisfaction= \beta_{1}Clinical supervision+\beta_{2}Feedback+\beta_{3}Workload+\beta_{4}Gender+\varepsilon$ [1]

$Trainee satisfaction= \beta_{1}Clinical supervision+\beta_{2}Feedback+\beta_{3}Workload+\beta_{4}Gender+\beta_{5}Supportive anvironment+\varepsilon$ [2]

Where:

$\beta_{1..n}$= regression coefficient

$\varepsilon$=error term

Check on linear OLS assumptions

Firstly, we estimated robust standard errors by using the Huber-White estimators (Table 4). From the analysis of the robust standard errors against the model ones, we can see that there are no major issues of failure to meet the linear OLS assumptions. Secondly, we ran a robust OLS regression. These show only minor problems with the OLS assumptions. Then, we checked for multi-co-linearity. Mean VIF value is lower than 2 (Table 4), therefore we can state that there are no issues of multi-co-linearity in our models.
